# A Computational Analysis of the Effect of Hard Choices on the Individuation of Values

**DOI:** 10.3390/brainsci15020131

**Published:** 2025-01-29

**Authors:** Norberto M. Grzywacz

**Affiliations:** 1 Department of Psychology, Loyola University Chicago, Chicago, IL 60660, USA; norberto@luc.edu; Tel.: +1-773-508-2970; 2 Department of Cognitive Science, Johns Hopkins University, Baltimore, MD 21218, USA

**Keywords:** cognition, choice consistency, decision-making, individuation of values, brain’s reward system, memory, Bayesian theory, surprisals, symmetry breaking

## Abstract

Background/Objectives: Experimental studies show that when an individual makes choices, they affect future decisions. Future choices tend to be consistent with past ones. This tendency matters in the context of ambivalent situations because they may not lead to clear choices, often leading people to make “arbitrary” decisions. Thus, because of choice consistency with the past, people’s decision-making values diverge. Thus, hard choices may contribute to the individuation of values. Methods: Here, we develop a Bayesian framework for the effects of cognitive choice consistency on decision-making. This framework thus extends earlier cognitive-science Bayesian theories, which focus on other tasks, such as inference. The minimization of total surprisals considering the history of stimuli and chosen actions implements choice consistency in our framework. We then use a computational model based on this framework to study the effect of hard choices on decision-making values. Results: The results for action selection based on sensory stimuli show that hard choices can cause the spontaneous symmetry breaking of the decision-making space. This spontaneous symmetry breaking is different across individuals, leading to individuation. If in addition, rewards are given to certain choices, then the direction of the symmetry breaking can be guided by these incentives. Finally, we explore the effects of the parametric complexity of the model, the number of choices, and the length of choice memory. Conclusions: Considering the brain’s mechanism of choice consistency and the number of hard choices made in life, we hypothesize that they contribute to individuality. We assess this hypothesis by placing our study in the context of the cognition-of-individuality literature and proposing experimental tests of our computational results.

## 1. Introduction

When an individual makes choices, they affect future ones because future decisions tend to be consistent with those from the past [1,2]. This consistency between past and future choices leads to the refinement or development of new preferences, a phenomenon referred to as “preference learning” [3]. In addition, this temporal consistency of choices has been shown to have important consequences for perception. For example, this consistency tends to lead to aesthetic stability [4]. Merely making a choice leads to a “spreading of alternatives”, whereby the two options become further apart in the preference domain, leading to improved detection [4]. Another consequence of choice consistency is increased confidence [5,6,7]. Self-consistency refers to an agreement between the current perceptual choice and the most frequent ones made for a given sensory stimulus and decision-making situation [7]. Perceptual confidence thus becomes an estimation of the probability that one would make the same decision given the same situation and physical stimulus. Consistently, emerging theories of decision-making and economics predict that as people gain experience with a decision-making task, their apparent internal decision noise decreases [8,9].

Surprisingly, however, although past choices tend to stabilize future ones, they may also contribute to the individuation of values. This tendency is especially important in the context of hard, ambivalent situations because they may not lead to clear choices, often leading people to make different, “arbitrary” choices. Thus, because of choice consistency with the past, people’s decision-making values may begin to diverge. The same does not happen in easy situations, that is, those for which choices are clear. In these situations, people have clear preferences on how to act, rarely making “arbitrary” decisions. Thus, preference learning may ensue only with hard choices. The role of preference learning for people’s individuality has been discussed elsewhere [10,11,12]. The emphasis of these studies has been on the reinforcement learning of aesthetic values of individuals under different exteroceptive (sensory) and interoceptive (body) environments [2,13,14]. Therefore, decision-making values would be learned to improve reward estimation, thus making it better. However, given the role of choices in preference learning, we must generalize these reward-based theories. The simplest way is to consider good past choices as rewards themselves. In this article, we propose a way to add past choices to the existing theoretical framework and analyze the consequences of the new emerging theory.

To add choice consistency to the current theoretical frameworks for preference learning, we must start with a deeper understanding of how they work. They have been proposed and analyzed in conditions of fixed [11,12] and varying [15] sensory environments. The basic idea of these frameworks is that at every instant, an individual receives sensory and body signals, and must decide on what action to take. A good decision depends on the correct estimation of reward for the various possible actions. After taking an action and receiving the reward, if the estimation is poor, the brain learns new parameters for its internal model of recompense expectation. In the case of aesthetic values, they are taken to be the expectation of reward. One can model such a process for aesthetic values with different mathematical tools. In this article, we take the approach of Bayesian theories of reinforcement learning [16,17,18]. These Bayesian theories have been especially useful in accounting for the process of inference [16]. Here, we expand them by adding choice consistency to the Bayesian framework by incorporating the recent history of the stimuli and decisions into the information provided to the individual. We assume that decisions made with choice consistency are more rewarding or equivalently, less discomforting.

Analysis of such theoretical frameworks has already shown that even without choice consistency, they tend to produce high individuality. Part of it appears from the exposure of individuals to the different statistics of distinct cultures and environments [12]. However, even if the statistics are similar, the stochasticity of the incoming signals causes variability in the values that we learn. This variability is magnified by a redundant space of values, that is, different parameters of the internal model of reward expectation predict the same rewards [11]. Our hypothesis is that choice consistency will magnify these individuation tendencies even further because random, hard decisions will separate people more.

In this article, we develop our Bayesian theoretical framework by adding to it the choice-consistency part. Bayesian theory has been influential in cognitive science, covering areas such as inference, perception, decision-making, and reinforcement learning [16,17,18,19,20,21,22,23,24]. The addition of choice consistency into the Bayesian framework allows for the possible emergence of cognitive individuation. We then use this expanded framework to develop a computational model to perform simulations. This model is rich enough to allow us to reach conclusions about how choice consistency may interact with reward mechanisms to contribute to individuality.

## 2. Theoretical Framework

We have split the description of the theory into two subsections, physical and mathematical. The “Physical Description” section (Section 2.1) has an account of the ideas without any equations. Our goal in that section is to help the reader understand the elements of the theory at an intuitive level. That section may allow readers to skip the equations (Section 2.2 and Section 3) at a first read and go directly to the “Results” (Section 4). We also place the mathematical developments in the appendices, leaving only the main equations in the “Mathematical Description” section (Section 2.2) to simplify the explanation.

### 2.1. Physical Description

**Figure 1:**
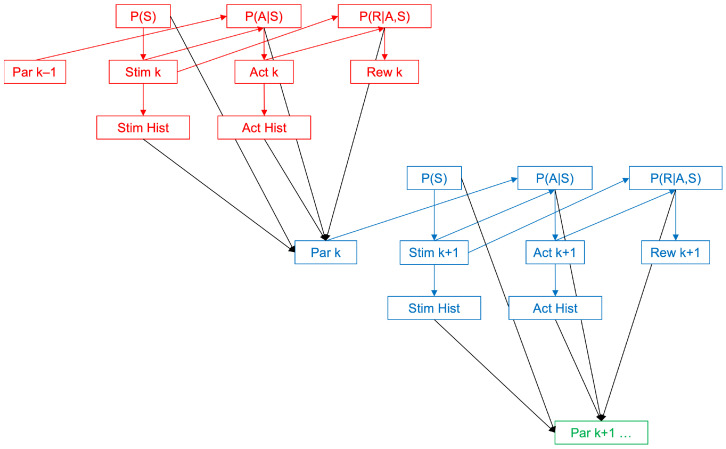
Bayesian update of the parameters of choice consistency. The figure shows three moments of parametric updates indicated in red, blue, and green. The first moment at time k (red) begins with a stimulus (Stim k) drawn from the probability distribution of stimuli (P(S)). With this stimulus, the brain calculates an action (Act k) from the probability distribution of actions given stimuli (P(A|S)). This calculation uses the set of parameters (Par k − 1) calculated at time k − 1. A reward (Rew k) then arrives from the probability distribution of rewards given stimuli and actions (P(R|A,S)). These stimulus and action are added to the histories of these values (Stim Hist and Act Hist). Given these histories and the new reward Rew k, a new parameter set (Par k) is computed, maximizing the Bayesian expected reward and action consistency. With this new set, one can repeat the process again at time k + 1 (blue). This process leads to the computation of a new parameter set (Par k + 1) that triggers the process again (green) and so on.

The core of a model of choice is that given information, for example, a sensory stimulus, the person must decide what action to take. The person uses a parametric internal model for this decision, with the parameters based on experience involving evolution (genes), development, learning, and choice consistency. How do the parameters change in time? In an optimal situation, parameter evolution should use a Bayesian process, recognizing that stimuli, choices, and rewards tend to be probabilistic. Figure 1 illustrates how the Bayesian update of parameters works.

Without loss of generality, we will mostly refer in this article to sensory stimuli because all experimental evidence so far has only addressed them. However, as explained in the next section, our theory is compatible with body signals as well. We will address their importance in the Discussion.

### 2.2. Mathematical Description

At time k, a person receives sensory and/or body signals and must decide what action $a_{k}$ to take. The best action must balance the maximization of reward, $r_{k}$, and the minimization of the deviation from past actions given similar stimuli. The stimulus $s_{k}$ is sampled from the probability distribution of inputs, $P\left(s\right)$ (Figure 1). From that, we use an internal model, $P\left(\left.a\right\rfloor s_{k},{\vec{w}}_{k-1}\right)$, to decide on the action to take, where ${\vec{w}}_{k-1}$ is the vector of parameters learned at time k−1. From the action sampled from the model, we can sample the reward $r_{k}$ from the probability distribution of rewards given actions and stimuli, $P\left(\left.r\right\rfloor {a_{k},s}_{k}\right)$.

In the next step, we update the parameters of the model before the next sampling begins. As mentioned above, this update must simultaneously optimize expected reward and consistency with past actions. So, we must perform an optimization that balances these two constraints. We do this by first finding the Bayesian expected loss for each constraint and then treating the problem as a Compromise Decision Problem [25,26].

The key decision in this step is the choice of parameters. To calculate the Bayesian expected choice-consistency loss as a function of the candidate parameters, we need the histories of the sampled stimuli and actions (Figure 1). We limit these histories to a finite number of time bins, that is, to a finite memory length. For each value of parameters, we can calculate the loss due to errors of choice consistency with these histories. The best parameters maximize the probability that from each stimulus in the past, we obtain the action seen in history. If we take the minus logarithm of this maximal probability, the best parameters minimize the total amount of surprisals in the history of the stimuli and actions. Thus, we choose the parameters that emphasize the most common stimulus–action pairs, weeding out the surprises. This choice yields the Bayesian expected choice-consistency loss $l_{a}\left({\vec{w}}_{k}\right)$. The value of the parameters also affects the Bayesian expected reward loss through the chosen action, and the probability distribution of rewards given actions and stimuli. We denote the reward loss as $l_{r}\left({\vec{w}}_{k}\right)$.

Finally, we propose working with Kempthorne’s λ-Bayes-based compromise problem [25] to propose that the brain minimizes

(1) $$l_{\lambda }\left({\vec{w}}_{k}\right)=\lambda l_{r}\left({\vec{w}}_{k}\right)+\left(1-\lambda \right)l_{a}\left({\vec{w}}_{k}\right),$$

where 0≤λ≤1. The parameter λ simply balances the competition between rewards and choice consistency in decision-making. Equation (1) and the parameter λ are like what Bayesian cognitive models of cue integration have proposed [27,28,29,30].

Any model based on Equation (1) must specify three mathematical quantities: $P\left(s\right)$, $P\left(\left.a\right\rfloor s,{\vec{w}}_{k}\right)$, $P\left(\left.r\right\rfloor a,s\right)$. To specify the three probability functions, we must begin with the domains of the variable. For simplicity, the stimulus and reward variables will be one-dimensional with an infinite domain, and we consider a discrete and finite action space. Furthermore, for simplicity, we base the probability distributions on Normal distributions. To begin, we take the distribution of the stimuli to be Normal, with zero mean and a given standard deviation $\sigma_{s}$:

(2) $$P\left(s\right)=\frac{e^{-\frac{s^{2}}{2\sigma_{s}^{2}}}}{\sqrt{2\pi }\sigma_{s}}.$$

Next, we represent the probabilistic relationship between stimulus and action as a sum of $N_{w}$ Normal distributions with a standard deviation $\sigma_{a}$, where the vector of parameters is ${\vec{w}}_{k}=\left(w_{k,1}^{\left(0\right)},w_{k,1}^{\left(1\right)},\ldots ,w_{k,N_{w}}^{\left(0\right)},w_{k,N_{w}}^{\left(1\right)}\right)$:

(3) $$P\left(\left.a\right\rfloor s,{\vec{w}}_{k-1}\right)=\frac{1}{N_{w}\sqrt{2\pi }\sigma_{a}}\sum_{i=1}^{N_{w}}e^{-\frac{{\left(a-\left(w_{k-1,i}^{\left(0\right)}+w_{k-1,i}^{\left(1\right)}s\right)\right)}^{2}}{2\sigma_{a}^{2}}}.$$

Hence, the components of the vector of parameters map linearly to the means of the Normal distributions selecting the actions. Finally, we also make the reward function a Normal distribution, standing for the probability of reward of each decision:

(4) $$P\left(\left.r\right\rfloor a,s\right)=\frac{1}{\sqrt{2\pi }\sigma_{r}}e^{-\frac{{\left(r-\alpha \left(a-\beta \right)*s\right)}^{2}}{2\sigma_{r}^{2}}},$$

where the parameters α and β set the relationship between the stimulus and the action to the mean reward for that pair, and $\sigma_{r}$ is the standard deviation.

## 3. Methods

### 3.1. Algorithm of the Computer Simulations

The simulation uses discrete time steps, labeled k. At time k, the model begins with the last estimation of the choice parameters, namely, ${\vec{w}}_{k-1}$. From this time, the simulations continue as follows:

- Sample $s_{k}$ (Equation (2)).
- Add $s_{k}$ to the history of stimuli (Appendix A.1).
- Sample $a_{k}$ (Equation (3)).
- Add $a_{k}$ to the history of rewards (Appendix A.1).
- Sample $r_{k}$ (Equation (4)).
- If not enough time has passed to accumulate enough history, go back to Step a.
- Otherwise, calculate ${\vec{w}}_{k}$ by minimizing Equation (1).
- Go back to Step a.

The minimization in Step g uses the simplex search method of Lagarias et al. [31].

### 3.2. Parameters of the Simulations

In this article, we report on simulations with different parameter sets to explore the model (see the end of Appendix A.2 for a complete list). We have chosen one of these sets as our standard set. We also show simulations with other parameter sets to illustrate individual differences and analyze the various behaviors of the model. Table 1 below shows the parameters of the standard simulations; we discuss others when presenting the results.

We have set λ=0 in this standard set because the emphasis of this article is on choice consistency, not rewards. Consequently, most simulations here are without the interference of rewards on the effects of choice consistency.

## 4. Results

### 4.1. Algorithm of the Computer Simulations

**Figure 2:**
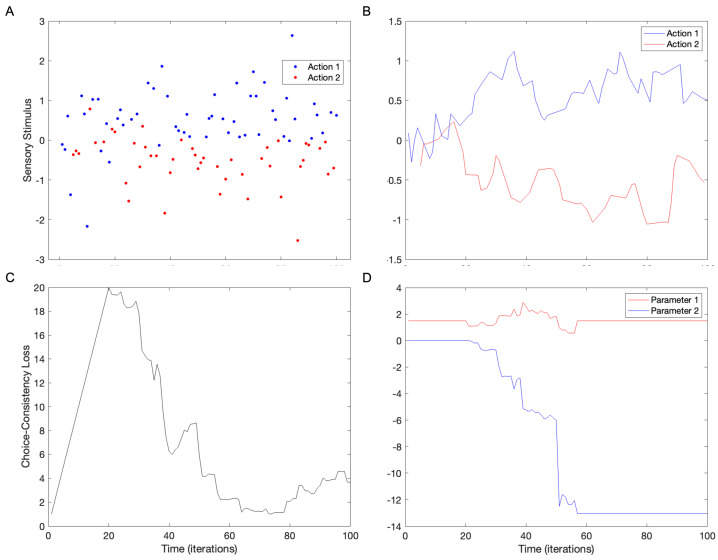
Computer simulation of our Bayesian theory of choice consistency with the standard parameters (Table 1). (**A**) Choices of two actions for different stimuli over time. An example of such an action is buying a shirt with this or that pattern. In this figure, every dot stands for a choice (color) for the given sampled stimulus at the given time. (**B**) Running average (5 points) of the choices in panel (**A**). (**C**) Choice-consistency loss as a function of time. (**D**) Temporal evolution of the two parameters of the model. These time courses reveal that the choices separate spontaneously, with an apparent phase transition in loss and parameters.

The goal of this article is to estimate whether people’s past choices may affect current ones significantly. The experimental observation is that choices of the past and present tend to be consistent. We have proposed a Bayesian theory for this consistency phenomenon. In this theory, choice consistency appears to adhere statistically to the recent history of stimuli and actions (Figure 1). Mathematically, such statistical adherence arises through the minimization of the total amount of surprisals in this history by judicial choice of the model parameters (Equation (1)). A typical computer simulation of a simple model based on such a total-surprisal theory of consistency appears in Figure 2.

The results of the computer simulations help us to understand how choice consistency develops over time. In the beginning, if choices are hard, they stay statistically split between the possible actions (Figure 2A). However, because the choices are initially random, a small statistical imbalance appears as a function of the stimuli. The proposed minimization of total surprisals then takes over, slowly expanding this imbalance and breaking the symmetry. In Figure 2A,B, this expansion causes Action 1 to occur mostly for positive stimuli and Action 2 for negative ones. This separation between the choices is initially slow but then reveals the characteristics of phase transition. A way to understand this phase transition is by seeing the total consistency loss (Figure 2C—Equation (3)). After a first climb resulting from the addition of stimuli and actions, the loss collapses, reaching a low plateau at around t=55. This collapse is accompanied by a sudden change in the second model parameter (Figure 2D). Other simulations yield comparable results, although the time of the phase transition and what parameters change can vary.

**Figure 3:**
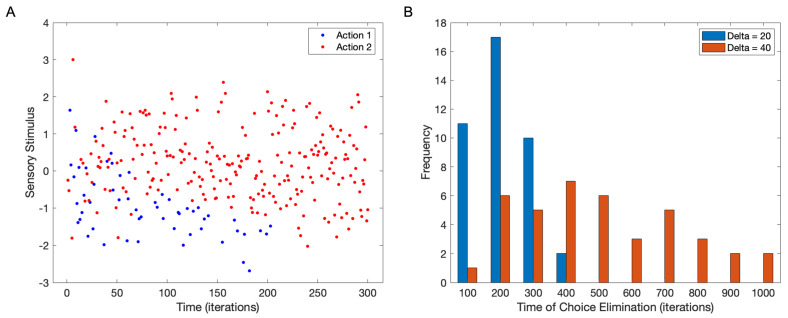
Elimination of choices. (**A**) Longer simulations with standard parameters (Table 1) show that eventually, choice consistency may cause one of the choices to eliminate the others. The conventions in this figure are the same as in Figure 2A. (**B**) Distribution of times of choice elimination for two values of memory length, namely, Δ.

How stable is the division of choices such as those in Figure 2? On one hand, one may expect major stability because after a division occurs it could self-perpetuate through the minimization of surprisals (Equation (1)). On the other hand, random sampling of choice could continue to bias one choice over another. This bias would then become stronger by symmetry-breaking mechanisms. Thus, one should not be surprised if eventually one choice swamps the other, taking over in perpetuity. Figure 3 assesses this idea by lengthening the simulation.

Figure 3A shows that with enough time, one choice may swamp the others. In this simulation, the equilibrium between the choices of Actions 1 and 2 persists until about t=30. After that time, choices of Action 2 for positive stimuli start breaking the symmetry. By t=100, most choices now go to Action 2, even in situations of slightly negative stimuli. Finally, after t=200, the choice of Action 1 is eliminated, with the individual always picking Action 2. With forty simulations as in Figure 3, Action 1 or Action 2 always wins out, and the mean time at which one of the actions disappears as a choice is 160±80 (standard deviation). Hence, the division of choices is not stable, and even with consistency, the brain may choose to drop some of them. The broad range of choice-disappearance times (Figure 3; blue bins) also implies individuality in the stability of decision-making based on the history of selections.

The length of memory (Δ) should influence stability and thus, the time when one choice swamps the other. One would expect that the longer this length is, the more stable the choice dependence on stimulus becomes. This stability should stem from the increased evidence for a particular discrimination of choices. One would expect that the stability would increase with the square root of Δ. Such dependence should hold because as a rule of thumb, the variability of the signal decreases by roughly the square root of the number of points averaged [32]. To test this prediction, we run the simulations with larger lengths of memory.

The results in Figure 3B show a significant choice-stabilization effect as the length of memory increases. The red bins (Δ=40) extend to longer times than the blue ones (Δ=20). The mean time when one choice takes over the other with Δ=40 is 450±240. This time is statistically significantly longer than that reported above for Δ=20 (one-sided *t*-test, t=5.32, 78 degrees of freedom, $p<{5\times 10}^{-7}$). The difference in stoppage times is ≈290 iterations despite Δ increasing by only 20. If one were to use the square root law theorized above, the increase in stoppage time by raising Δ by a factor of 2 should have been only $160*\sqrt{2}-160\approx 70$. Consequently, the effect of Δ on stoppage time is much more than simply increasing sampling. In contrast, the percentage of time in which the full discrimination of actions by stimuli occurs is 73%±7% for Δ=40. This percentage is not statistically significantly different from that seen for Δ=20.

### 4.2. Individuation of Values from Choice Consistency

**Figure 4:**
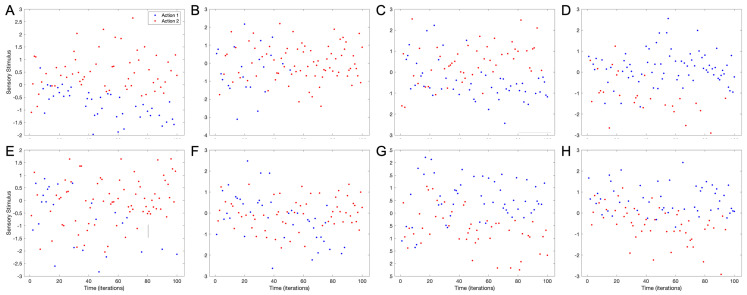
Eight consecutive simulations of actions in response to sensory stimuli with the standard set of parameters, using the conventions of Figure 2A. Each simulation yielded a unique pattern of behavior. After a first random phase, the most common behaviors were such that positive sensory stimuli tended to yield Action 1 (Panels **D**,**G**,**H**) or Action 2 (Panels **A**,**C**,**E**). In these behaviors, negative sensory stimuli tended to yield the opposite actions. Occasionally, we also saw a behavior that was more mixed (Panel **B**). More rarely, we saw a behavior in which an action happened for positive stimuli earlier and negative ones later (**F**).

The spontaneous symmetry breaking with choice consistency revealed in Figure 2 and Figure 3 suggests the possibility of another surprising phenomenon. Given that spontaneous symmetry breaking starts with small statistical imbalances, if these were distinct for different individuals, they may display completely different patterns of choices over time. Thus, random first hard choices may lead to later individuation even for identical observers. To evaluate this individuation-by-choice idea, we repeated the simulations of Figure 2 and Figure 3 multiple times. The results of these simulations and their implications for individuation appear in Figure 4.

Figure 4 shows that the symmetry breaking induced by choice consistency can lead to strong individuality. This individuality expresses itself in multiple forms. The most usual form (80%±6% in forty simulations; standard error) is that positive stimuli eventually give rise to Action 1 (Figure 4D,G,H) or Action 2 (Figure 4A,C,E), and vice versa for negative stimuli. Another form is that the chosen actions stay intermingled until one of them takes over (Figure 4B—12%±5%). Finally, an action may occasionally correspond to positive stimuli early and negative ones later (Figure 4F—8%±4%). Another important observation is that the time of separation of actions by stimuli varies, occurring early (Figure 4A) or late (Figure 4D). Therefore, early choices can cause strong individuality that may remain for a long time.

Another key observation is that the stimuli for which the model makes a choice are not constant but drift continuously. As mentioned above, one can see this in Figure 4F. However, even when the order of the stimuli yielding an action does not change, one can see a drift in the space of choices. For example, in Figure 4A,C, the stimuli giving rise to Action 1 become increasingly negative. Consequently, the selection of actions under choice consistency shows temporal instability.

**Figure 5:**
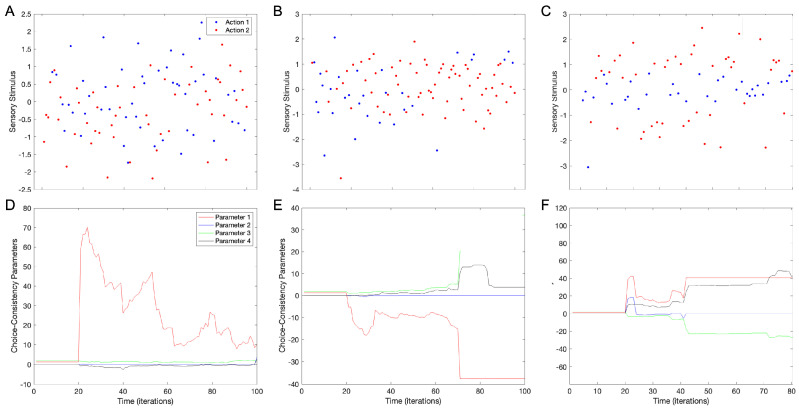
Increasing the number of choice parameters boosts the individuality arising from the model. In these simulations, we substituted ${\vec{w}}_{0}=\left(1.25,0,1.75,0\right)$ for the standard value in Table 1 and thus, we had four choice parameters instead of two. (**A**–**C**) Examples of choices (with conventions as in Figure 2A). (**D**–**F**) Temporal evolution of parameters in the simulations of (**A**, **B**, and **C**) respectively. Most simulations with 4 choice parameters yielded behaviors like those in Figure 4. However, some simulations yielded different behaviors, as illustrated in this figure. (**A**,**D**) Examples of not discriminating actions by stimuli. (**B**,**E**) Examples of switching stimulus dependence of choices. (**C**,**F**) Examples of Action 1 sandwiched between two stimulus locations of Action 2.

We have also reasoned that the individuation may become more pronounced if the number of choice parameters (${\vec{w}}_{k}$—Equation (3)) increases. The logic is that with more parameters, the set of choices becomes larger. This logic follows from a recent study showing that parametric redundancy increases individuality when performing reinforcement learning of aesthetic values with stochastic inputs [11]. Alternatively, an increase in the number of parameters may instead lead to more chaos and thus, apparently less organized choices. Figure 5 shows a test of these alternatives. This test uses standard parameters (Table 1), except that we have four choice parameters (${2\times N}_{w}=4)$ instead of two (${2\times N}_{w}=2)$.

The increase in the number of choice parameters from two to four boosts individuation by adding new choice behaviors. Most simulations (out of forty) still give behaviors as in Figure 2, Figure 3 and Figure 4 (70%±7%). However, three new behaviors appear with more parameters. The first, and the rarest, is the continued intermingling of Actions 1 and 2 regardless of the stimuli (2.5%±2.5% of the simulations—Figure 5A). The second is cases in which one choice shifts to different stimuli during the simulation (7.5%±4% of the simulations). Thus, in Figure 5B, Action 2 tends to occur with negative stimuli until about t=60, but then shifts to positive ones after that. Finally, the third is a behavior in which a choice at intermediate stimuli (≈0 in Figure 5C) has the other choice for higher and lower stimuli (≈−1 and ≈1 in Figure 5C). We have even seen such a sandwich behavior with four bands, for example, Action 1, Action 2, Action 1, and Action 2, as the stimulus increases. This sandwich behavior happens in 20%±6% of the simulations.

This increase in individuation can be understood in terms of the larger variability of the choice parameters. The intermingling, non-discriminating behavior in Figure 5A results from the dominance of one of the parameters over the others (Figure 5D). Such dominance reduces the ability to make discriminating choices. In turn, the sudden shift in the choice of Action 2 in Figure 5B results from a phase transition in three of the four parameters (Figure 5E). Finally, the sandwich behavior (Figure 5C) arises from a delicate balance between the four choice parameters (Figure 5F). Such richness of behaviors is not possible with just two choice parameters because they have a more limited repertoire (Figure 2D).

### 4.3. The Effect of the Number of Choices

**Figure 6:**
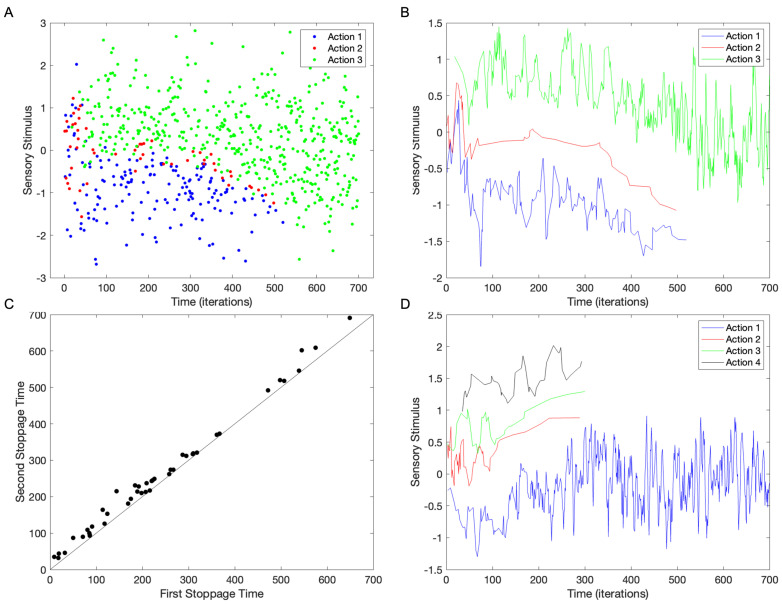
Outcome of the simulations with three or four choices instead of two. (**A**) Choices of three actions for different stimuli over time (conventions as in Figure 2A). (**B**) Running average (5 points) of the choices in Panel (**A**). These panels show that choice consistency organizes the three actions in the space of stimuli. However, eventually one action dominates (Action 3 in this example), with one of the other actions stopping first (Action 2 in this example) and then the other (Action 1). (**C**) Scatter plot of the stoppage times of the losing actions. They tend to stop almost at the same time. (**D**) Running average (5 points) of a simulation with four choices.

So far, the simulations have focused on two choices ($N_{a}=2$) because that is the most common situation in laboratory settings [7]. However, in the real world, the number of choices is often larger. We thus asked in what ways this number would affect the results. Our first prediction was that in a constrained space of stimuli, we could not achieve full discrimination of choices when the number of actions became high. Figure 6 shows a test of this prediction by using standard parameters (Table 1), except that we have more than two choices ($N_{a}>2$).

The results show that with the standard parameters, except for three choices ($N_{a}=3$), the model can segment the actions into different regions of the stimulus space (Figure 6A,B). In forty simulations, such three-way segmentation happens often (68%±7%). Hence, the incidence of three-way segmentation is not statistically significantly different from that seen with two choices. When the segmentation does not happen, we see either the intermingling of choices (as in Figure 4B; 25%±7%) or a two-way segmentation with two choices combined (7%±4%). When the three-way segmentation happens, one of the actions comes to dominate eventually (Figure 6A,B), as for two choices (Figure 3). The stoppage times of the two losing actions are almost identical (Figure 6C). Plotting the second stoppage time against the first reveals an almost perfect correlation (r=0.996±0.003), with linear regression giving an intercept of 21±4 (standard error) and a slope of 1.00±0.01. Therefore, the two stoppage times are about a constant 20 iterations apart. Moreover, these two stoppage times tend to occur later than when the stimulations had two choices (Figure 3). The first stoppage time with three choices is at 220±160, while the second is at 240±150.

One of the most interesting results in Figure 6A is that the stimulus range for Action 2 is narrow. In all simulations, the actions sandwiched between the other two have a narrow range. This narrowness is not surprising because different from the outer actions, the inner one has little room to expand. The narrowness of the inner action allows us to inspect in more detail the instability of the choices. In Figure 4, the instability appears as choices corresponding to stimuli that are more negative over time. However, the narrowness of the range of stimuli of Action 2 in Figure 6A,B reveals that the instability can show richer behavior. In these figures, the stimuli yielding Action 2 rise slowly initially and then fall rapidly. In multiple simulations, we have seen different rise-and-fall behaviors for the inner action.

We can still achieve full discrimination of actions by stimuli when the simulations run with more than three choices ($N_{a}>3$—Figure 6D). However, full discrimination becomes rarer as the number of choices increases. With forty simulations, the percentage of time to achieve full discrimination falls to 53%±5% with four choices ($N_{a}=4$). This percentage collapses with five choices or more, becoming 10%±5% with $N_{a}=5$ and 0% with $N_{a}=6$. We do not achieve any full discrimination with six choices or more ($N_{a}>6$) with our standard parameters. This lack of discrimination is consistent with our first prediction that in a constrained space of stimuli, one cannot achieve full discernment of choices when the number of actions is high.

### 4.4. The Interaction Between Reward and Choice Consistency

**Figure 7:**
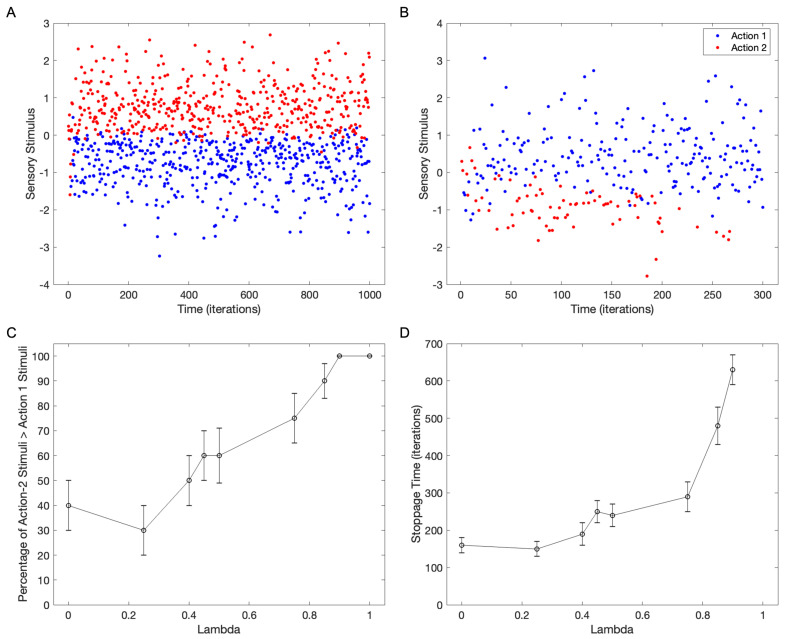
The interaction between rewards and choice consistency with standard parameters, except for variations of λ. (**A**) Simulation with only rewards (λ=1). As expected from the choice of the standard parameters, Action 2 is chosen for positive stimuli and vice versa for Action 1. (**B**) Example of simulation with λ=0.5 in which positive stimuli elicit Action 1 despite the rewards favoring the opposite. (**C**) Percentage of simulations for which Action 2 stimuli converge to values larger than those for Action 1 as a function of λ. (**D**) Mean stoppage time of the losing actions as a function of λ. Error bars in (**C**,**D**) are standard errors. As λ increases, we obtain more Action 2 because of the rewards, and the stoppage time rises because the influence of choice consistency diminishes.

The simulations have focused so far on choice consistency. However, in the real world, choices can be affected by both their consistency and their elicited rewards [11,12,15]. We expect that if a reward favors the relation relationship between certain stimuli and an action, then this incentive would tend to bias the final discrimination. Consequently, we expect a reduction in the contribution to individuation by choice consistency. This reduction would not mean less total individuality because rewards can cause individuation by themselves [11,12]. To test this predicted competition between rewards and choice consistency, we use the standard parameters, except for varying the value of λ, the parameter weighing the impact of these two factors (Figure 7). The design of the standard parameters is such that Actions 1 and 2 will obtain rewards for negative and positive stimuli, respectively.

Figure 7A shows that if only rewards affect the choices (λ=1), then they will be such as to maximize the rewards. For the standard parameters, this maximization means that negative and positive stimuli will yield Action 1 and Action 2, respectively. Furthermore, different from what happens with choice consistency alone, no action dominates the other even after a long time. However, as λ falls, the percentage of simulations for which action-2 stimuli converge to values larger than those for Action 1 decreases (Figure 7B,C). This curve has a sigmoidal shape as a function of λ, with the change in dominance from Action 1 to Action 2 occurring between λ≈0.4 and λ≈0.9 for the standard parameters. Another important result is that the stoppage time of the losing action increases with λ (Figure 7B,D). For the standard parameters, the stoppage time curve rises rapidly after about λ=0.5. This rise tends towards infinity because no action ever stops for λ=1. Hence, in the competition between reward and choice consistency, the model picks the action as a hybrid between them.

## 5. Discussion

### 5.1. Contribution to Individuation by Symmetry Breaking in the Minimization of Surprisals

We have proposed that the brain implements choice consistency by a minimization of the sum of surprisals [33]. Thus, we propose minimizing unexpected actions given both the current stimulus and the history of stimuli and actions. Such a minimization has been used in other cognitive-science contexts, such as in perception [34,35] and active inference [36]. Other models are possible for choice consistency, of course, but minimization of surprisal appears naturally from the Bayesian framework. We will discuss these alternate models below.

Our results show that such a surprisal-minimization mechanism of choice consistency can contribute to the individuation of values. Individuality means variance within and across people [37]. Consequently, because individuality means that different people are distinct and because the simulations all have the same initial conditions, the individual uniqueness implies that the system undergoes spontaneous symmetry breaking [38,39,40]. In physical systems, spontaneous symmetry breaking implies equations of motion obeying symmetries, as well as a lowest-energy state without the same symmetries. Our equations of motion are symmetric in that stochastic sampling allows any action to connect with any stimulus. However, when a connection between certain neighbor stimuli and an action becomes statistically stronger than others, the minimization of surprisals makes future choices compatible with this link, reinforcing it. This is positive feedback because this reinforcement makes the connection itself stronger. Such positive feedback is not unique to surprisal-minimization theories. But whatever the theory used, choice consistency will reinforce the connection between certain past stimuli and an action. Such a spontaneous symmetry breaking of choices with positive feedback is bound to generate phase transitions [39]. We see phase-transition behavior in how the choice parameters change in our simulations.

Another result is that increasing the number of choice parameters strengthens the individuation tendency. Parametric redundancy has been studied recently as a way to increase system reliability [41]. Such redundancy allows the automatic re-assignment of tasks performed by a basic element to a backup one. From our perspective, redundancy allows for the generation of optimal parametric surfaces instead of points [42]. Therefore, because solutions can exist at different points on the surfaces, we can achieve individuality. As such, the individuation of values achieved with our minimal-surprisal theory of choice consistency has a relation to that achieved with reinforcement learning of rewards, which also has parametric redundancy [11].

### 5.2. Why a Choice Always Eventually Dominates

We have already seen that spontaneous symmetry breaking causes different chosen actions to occupy distinct portions of the stimulus space. However, spontaneous symmetry breaking has another effect, namely, in every simulation, a choice ends up dominating the others after enough time has elapsed. To understand this effect, let us begin with the simplest example, that of Figure 3A. In this example, the model initially samples the action probabilistically. With the initial condition of the choice parameters, the mean of the underlying probability distribution is exactly in the middle between the two possible actions. However, over time, the mean moves statistically in the direction of one of the actions. This movement causes the parameters to shift towards the action, producing positive feedback. It, in turn, causes a phase transition that ends up dropping the alternate action. Its removal is a form of spontaneous symmetry breaking because the equations of motion do not favor any action in the beginning.

This spontaneous symmetry-breaking process can also account for two features of the times at which the losing actions stop: First, the stoppage times have a broad range. Such a range occurs because the model is stochastic and must reach the phase transition point. The distribution of first-passage times through this point is broad when the stimuli do not have bounds, as in our case [43,44,45]. Second, when more than two choices are available, the losing actions tend to die at similar times. The positive-feedback process for choice elimination explained in the last paragraph works here as well. As the parameters move towards the winning choice, the process reinforces the motion itself in a positive-feedback loop. Hence, the winning choice becomes stronger, ending the others quickly in a phase transition.

### 5.3. How to Stabilize the Effect of Choice Consistency

Can one change the theory or model to stabilize choices such that actions do not stop after a while? From the simulations, the strongest factor stabilizing the actions is the length of memory. Consequently, a way to stabilize the choices is to make the length of memory infinitely long. Of course, the brain cannot remember all the pairs of choices and stimuli in the distant past. However, an alternative is to accumulate past choices by using them to adjust the parameters of a model, such as in reinforcement learning [46,47]. Such adjustment can consider as much of the past as one wishes.

We emphasize that the instability is in part due to the use of a continuous stimulus space. In the laboratory, the number of stimuli is typically finite [5,6,7]. With a finite set of well-separated stimuli, an action cannot easily invade the space of another one. This separation then results in more stability that we see in our simulations. Future experiments with longer durations and/or with continuous stimulus variables can test the instability predictions of this article.

### 5.4. How to Get More Choices Represented

With our model and the standard parameters, only up to five choices could fully be discriminated by the stimuli eliciting them. Can we change the theory or the model to allow for the discrimination of more choices? The simplest such change is the reduction in the standard deviation of the connection between stimuli and choices. Such a reduction would separate the actions more with respect to the stimuli. Another way to improve the discrimination of actions is to use a nonlinear model for the relationship between stimuli and the mean of the distribution. If well designed, such a nonlinearity could allow a better separation between an action and its neighbor. Finally, the model could use a non-Normal distribution for the stimuli to counter the tendency of Normal distributions to bunch up the outputs around the mean. Instead, leptokurtotic distributions tend to have fatter, longer tails, allowing for more discrimination of actions [48]. Such distributions occur in natural [49,50,51] and human-made environments [52].

### 5.5. Reward Versus Choice Consistency

The competition between reward and choice-consistency mechanisms in the context of individuation merits a discussion. Reward mechanisms have been shown to elicit individuation in at least three ways: First, reinforcement learning of prediction of reward leads individuals from diverse cultures to develop different values [12,53,54]. Second, interoceptive (body) inputs to the brain modulate the learning of aesthetic values in ways that are individual [12,13]. Third, the parametric redundancy intrinsic to reinforcement learning of rewards leads to individuation (Section 5.1). In this article, we show that parametric redundancy also helps magnify the individuality generated by choice consistency. And although we have emphasized sensory stimuli, the theory for choice consistency is also compatible with interoceptive signals (Section 2). This compatibility adds further individuation power to choice-consistency mechanisms.

The question for us is as follows: are reward and choice mechanisms for individuation independent, adding to each other linearly, or do they interact in a nonlinear way? Our results show that this interaction is nonlinear. The more the model considers rewards, the more biased the choices become, to the point of occasionally disregarding consistency with the past. Thus, if choice consistency is akin to avoiding cognitive dissonance, we may avoid it if the price is right. However, we can also see this competition from the opposite perspective to reach a well-known startling conclusion. People often have so much discomfort with cognitive dissonance that they may make choices that are irrational from a reward (utility) perspective [55,56,57]. To bring back the discussion to individuality, different people have distinct degrees of aversion to cognitive dissonance [58,59,60]. Such a difference in degrees of aversion implies an inter-individual variability in the weights that people use to balance reward versus choice consistency. Therefore, although rewards may reduce the individuation due to choice consistency, the competition between these factors may vary across individuals.

### 5.6. Practical Applications

The results described in this article on the effects of rewards and choice consistency may have practical applications for society. The next section will address implications for cognitive psychology, while here, we provide three examples of real-world applications: First, in the marketing and behavioral economics fronts, rewards would bias initial consumer choices that would then linger for a long time [3,8,9,61,62,63]. Second, choice consistency has been used in clinical decision-making [64], especially for individuals with intellectual and developmental disabilities [64]. Third, what will happen when we start producing artificial intelligence (AI) systems that learn autonomously? Currently, such systems depend on their programmers. However, imagine, for example, an AI system in a spaceship exploring outer space. Because in these situations, one does not know what one may find, perhaps these systems should make their own choices, learning from them. And as for humans on Earth, perhaps choice consistency and free reinforcement learning may make sense as a part of how these systems learn. A consequence of this learning will be the breaking of symmetry and individuation of these AI systems.

### 5.7. Relationship with Other Studies of Individuation and Experimental Predictions

The brain has mechanisms to allow the individuation of cognitive values to continue throughout life [65,66,67,68]. They are complex and not compatible with trait models of individuality [69]. Thus, these mechanisms clash with the idea that personality is made up of stable characteristics that influence how people think, feel, and behave in different situations [70,71,72]. Instead, individuation is associated with value instability [3,4]. In this article and elsewhere, we propose that these differences are due in part to brain mechanisms of stochastic learning [11,12,73]. The brain valuation system has been extensively studied [74] and the involvement of reward-learning systems, such as the basal ganglia, has been established [13]. Such reward-learning systems may also help in learning choice consistency. This is because choice inconsistency may be related to cognitive dissonance (Section 5.5), which can be viewed as a negative reward. Other areas involved in choice inconsistency include the ventromedial prefrontal cortex, anterior cingulate cortex, and posterior cingulate cortex [75]. Another result of interest related to the cognition of choice consistency is that taxing cognitive capacities reduces choice consistency [76]. An explanation for this reduction is that under cognitive taxation, people make impulsive, irrational choices [76,77,78]. Alternatively, people change their decision strategies to simpler ones when the cognitive load is higher [76,79].

This discussion on the cognitive mechanisms of choice consistency suggests new experiments to assess the computational models of this article. In these experiments, we imagine subjects first performing a continuous Likert rating [80] of multiple stimuli organized according to a variable (for example, degree of complexity). Afterwards, we perform two alternative forced choices of pairs of these stimuli, especially those similarly liked during the Likert rating. We then return later to the Likert rating to see if the forced choice breaks the symmetry. Moreover, we repeat the rating days or weeks later to evaluate if the individuality is “permanent”. This general rating technique allows us to answer other questions. For example, does taxing cognitive capacities affect the learning of the choices or just the later decisions? Similarly, does taxing cognitive capacities cause impulsive choices or a simplified decision strategy? To answer these questions, we can repeat the later measurements to study whether the choices are inconsistent with the past but internally consistent. Finally, we can use modified versions of these kinds of measurements to evaluate the interaction between reward and choice consistency.

### 5.8. Do Hard Choices Have an Effect on the Individuation of Values?

In this section, we go back to the issue raised by the title of the article, namely, “the Effect of Hard Choices on the Individuation of Values”. To start, let us consider what hard choices are. They emerge in four main situations [81,82]: First, one often makes choices with incomplete information. Second, hard choices often arise from complex challenges, that is, those for which calculating an ideal action is difficult. Third, hard choices also arise from incomplete preferences, often resulting from novel situations, for which people have not yet developed clear values. Research has shown that behavior that is indicative of incomplete preferences is empirically associated with deliberate randomization as we use in our simulations [83]. Fourth, when making choices, people must regularly juggle competing values, and no decision satisfies them all. Sometimes choice conflicts arise between one’s important values, causing negative emotions [84]. Such negative emotions can contribute to perceived decision difficulty [85,86,87].

Do these four situations occur often enough in everyday life to make hard choices significant factors in the individuation of values? According to some estimates, a person makes thousands of choices a day [88,89]. These choices include, for example, what to eat [90], what to wear [91], and when to slow down when driving a car [92]. Such choices are often hard because of one or more of the four situations described above. For example, making choices with incomplete information is common in tasks [62,93,94].

Therefore, given that hard choices are common, we can now address the issue in the title of this article. We begin by admitting that one cannot fully clarify this issue without experimentation (but see Section 5.7). However, the theoretical work described here strongly suggests that the answer is yes. Our theoretically optimal implementation of choice consistency leads us to believe that making hard choices makes our values diverge from those of others. Hence, we argue that hard choices contribute to our individuality.

## Figures and Tables

**Table 1:** Standard set of parameters.

Parameter(s)	Equation	Values
${\vec{w}}_{0}$	1	$\left(1.5,0\right)$
$N_{w}$	3	1
$N_{a}$	A8	2
Δ	A2	20
λ	1	0
$\sigma_{a}$	3	1
$\sigma_{r}$	4	1
$\left[\alpha ,\beta \right]$	4	$\left[2,3/2\right]$

## Data Availability

The original contributions presented in this study are included in the article. Further inquiries can be directed to the author.

## Appendix A

### Appendix A.1. A Bayesian Description of the Framework

At time k, a person receives sensory and/or body signals $s_{k}$, and must decide what action $a_{k}$ to take. The best action must balance the maximization of reward, $r_{k}$, and the minimization of the deviation from past actions given similar stimuli. The stimulus $s_{k}$ is sampled from the probability distribution of inputs, $P\left(s\right)$ (Figure 1). From that, we use an internal model, $P\left(\left.a\right\rfloor s_{k},{\vec{w}}_{k-1}\right)$, to decide on the action, $a_{k}$, to take. For this model, we use the vector of parameters ${\vec{w}}_{k-1}$ learned at time k−1. From the $a_{k}$ outputted by the model, we can sample the reward $r_{k}$ from the probability distribution of rewards given actions and stimuli, $P\left(\left.r\right\rfloor {a_{k},s}_{k}\right)$. The presence of $s_{k}$ in this probability distribution follows the same rationale given elsewhere that the reward depends on both the stimulus and the action taken [11,12].

In the next step, we update the parameters of the model before the next sampling begins. As mentioned above, this update must simultaneously optimize expected reward and consistency with past actions. So, we must perform an optimization that balances these two constraints. We do this by first finding the expected loss for each constraint and then treating the problem as a Compromise Decision Problem [25,26].

The key decision in this step is the choice of ${\vec{w}}_{k}$. To calculate the expected choice-consistency loss as a function of the candidate parameter $w_{k}$, we need the histories of the sampled stimuli and actions (Figure 1). We denote the number of time bins used in the histories as Δ. Therefore, Δ stands for how long the memory of choices last. For each value of $w_{k}$, we can calculate the loss due to errors of choice consistency with these histories. The best $w_{k}$ maximizes the probability that from each $s_{i}$ we obtain the $a_{i}$ observed in history, that is, maximizes $\prod_{i=k+1-\Delta }^{k}P\left(\left.a_{i}\right\rfloor s_{i},{\vec{w}}_{k}\right)$. If we take the minus logarithm of this quantity, the best ${\vec{w}}_{k}$ obeys

(A1) $${\vec{w}}_{k}\left({\overset{ˇ}{s}}_{k},{\overset{ˇ}{a}}_{k}\right)={\mathrm{argmin}}_{{\vec{w}}^{*}}-\sum_{i=k+1-\Delta }^{k}\log_{2}\left(P\left(\left.a_{i}\right\rfloor s_{i},{\vec{w}}^{*}\right)\right),$$

where, for simplicity, we denote the histories with the notation [95,96,97,98]

(A2) $${\overset{ˇ}{x}}_{k}=\left(x_{k+1-\Delta },\;\cdots ,x_{k}\right)$$

Equation (1) has an important interpretation because the −log terms are the surprisals of $a_{i}$ given $s_{i}$ [33]. Consequently, the optimal ${\vec{w}}_{k}$ minimizes the total amount of surprisals in the history of the stimuli and actions. Thus, we choose the ${\vec{w}}_{k}$ to emphasize the most common ($s_{i}$, $a_{i}$) pairs, weeding out the surprises. This choice yields the expected choice-consistency loss as

(A3) $$l_{a}\left({\vec{w}}_{k}\right)=-\sum_{i=k+1-\Delta }^{k}\log_{2}\left(P\left(\left.a_{i}\right\rfloor s_{i},{\vec{w}}_{k}\right)\right).$$

Because we sample $a_{i}$ and $s_{i}$ according to their own probability, we can write the corresponding loss function [99,100]

(A4) $$L_{a}\left(s,a,{\vec{w}}_{k}\right)={-\log}_{2}\left(P\left(\left.a\right\rfloor s,{\vec{w}}_{k}\right)\right),\;$$

namely, the surprisal itself.

The value of ${\vec{w}}_{k}$ also affects the expected reward loss through the chosen action and the function $P\left(\left.r\right\rfloor a,s\right)$. The expected reward is $∭rP\left(\left.r\right\rfloor a,s\right)P\left(\left.a\right\rfloor s,{\vec{w}}_{k}\right)P\left(s\right)ds\;da\;dr$. The reward loss is then

(A5) $$l_{r}\left({\vec{w}}_{k}\right)=-∭rP\left(\left.r\right\rfloor a,s\;\right)P\left(\left.a\right\rfloor s,{\vec{w}}_{k}\right)P\left(s\right)ds\;da\;dr.$$

Again, because the decision is on $w_{k}$, the expectation is through $P\left(s\right)$, making the loss function

(A6) $$L_{r}\left(s,w_{k}\right)=-\int rP\left(\left.r\right\rfloor s,{\vec{w}}_{k}\right)dr\;$$

after integrating by a. The interpretation of this loss function is simple: find the parameter that increases the reward for the expected stimuli.

Finally, we propose working with Kempthorne’s λ-Bayes-based compromise problem to propose that the brain minimizes

(A7) $$l_{\lambda }\left({\vec{w}}_{k}\right)=\lambda l_{r}\left({\vec{w}}_{k}\right)+\left(1-\lambda \right)l_{a}\left({\vec{w}}_{k}\right)\;,\;$$

where 0≤λ≤1. See Kempthorne’s theorems 3.1 and 3.2 describing the properties of the λ-based compromise problem [25].

### Appendix A.2. A Simple Model to Explore the Properties of the Theory

Any model based on Equations (1)–(7) must specify six mathematical quantities: $P\left(s\right)$, $P\left(\left.a\right\rfloor s,{\vec{w}}_{k}\right)$, $P\left(\left.r\right\rfloor a,s\;\right)$, $N_{w}$, Δ, and λ. To specify the three probability functions, we must begin with the domains of s, a, and r. For simplicity, the stimulus and reward variables will be one-dimensional, such that s,r∈R, and we consider a discrete and finite action space (with $N_{a}$ possible actions) represented as

(A8) $$a\in \left(1,2,\;\ldots ,\;N_{a}\right)\;.\;$$

Again, for simplicity, we base $P\left(s\right)$, $P\left(\left.a\right\rfloor s,{\vec{w}}_{k}\right)$, and $P\left(\left.r\right\rfloor a,s\;\right)$ on Normal distributions. To begin, we take the distribution of s to be Normal, with zero mean and a given standard deviation $\sigma_{s}$:

(A9) $$P\left(s\right)=\frac{e^{-\frac{s^{2}}{2\sigma_{s}^{2}}}}{\sqrt{2\pi }\sigma_{s}}\;.\;$$

Next, we represent the probabilistic relationship between s and a as a sum of $N_{w}$ Normal distributions, where ${\vec{w}}_{k}=\left(w_{k,1}^{\left(0\right)},w_{k,1}^{\left(1\right)},\ldots ,w_{k,N_{w}}^{\left(0\right)},w_{k,N_{w}}^{\left(1\right)}\right)$ with equal standard deviation $\sigma_{a}$:

(A10) $$P\left(\left.a\right\rfloor s,{\vec{w}}_{k-1}\right)=\frac{1}{N_{w}\sqrt{2\pi }\sigma_{a}}\sum_{i=1}^{N_{w}}e^{-\frac{{\left(a-\left(w_{k-1,i}^{\left(0\right)}+w_{k-1,i}^{\left(1\right)}s\right)\right)}^{2}}{2\sigma_{a}^{2}}}\;.\;$$

Hence, the components of ${\vec{w}}_{k}$ map linearly to the means of the Normal distributions, selecting the actions. Finally, we also make the reward function a Normal distribution, standing for the probability of reward of each possible decision:

(A11) $$P\left(\left.r\right\rfloor a,s\right)=\frac{1}{\sqrt{2\pi }\sigma_{r}}e^{-\frac{{\left(r-\alpha \left(a-\beta \right)*s\right)}^{2}}{2\sigma_{r}^{2}}}\;,\;$$

where the parameters α and β set the relationship between the stimulus s and the action a to the mean reward for that pair, and $\sigma_{r}$ is the standard deviation.

To summarize, the parameters that control the outcome of the simulations of the model are ${\vec{w}}_{0}$, $N_{w}$, $N_{a}$, Δ, λ, $\sigma_{s}$, $\sigma_{a}$, $\sigma_{r}$, α and β. Without loss of generality, we can set $\sigma_{s}=1$ and control the simulations with the rest of the parameters. Thus, we have nine parameters with which to explore the predictions of the theory.
